# Interrogation Method with Temperature Compensation Using Ultra-Short Fiber Bragg Gratings in Silica and Polymer Optical Fibers as Edge Filters

**DOI:** 10.3390/s23010023

**Published:** 2022-12-20

**Authors:** Luís Pereira, Rui Min, Getinet Woyessa, Ole Bang, Carlos Marques, Humberto Varum, Paulo Antunes

**Affiliations:** 1 I3N & Physics Department, University of Aveiro, 3810-193 Aveiro, Portugal; 2 Center for Cognition and Neuroergonomics, State Key Laboratory of Cognitive Neuroscience and Learning, Beijing Normal University, Zhuhai 519087, China; 3 DTU Electro, Department of Electrical and Photonics Engineering, Technical University of Denmark, 2800 Lyngby, Denmark; 4 CONSTRUCT-LESE, Structural Division, Department of Civil Engineering, Faculty of Engineering, University of Porto, 4200-465 Porto, Portugal; 5 Instituto de Telecomunicações, University of Aveiro, 3810-193 Aveiro, Portugal

**Keywords:** fiber Bragg grating, optical edge filter, polymer optical fiber, strain sensor, temperature compensation

## Abstract

The use of simpler and less bulky equipment, with a reliable performance and at relative low cost is increasingly important when assembling sensing configurations for a wide variety of applications. Based on this concept, this paper proposes a simple, efficient and relative low-cost fiber Bragg grating (FBG) interrogation solution using ultra-short FBGs (USFBGs) as edge filters. USFBGs with different lengths and reflection bandwidths were produced in silica optical fiber and in *poly(methyl methacrylate)* (PMMA) microstructured polymer optical fiber (mPOF), and by adjusting specific inscription parameters and the diffraction pattern, these gratings can present self-apodization and unique spectral characteristics suitable for filtering operations. In addition to being a cost-effective edge filter solution, USFBGs and standard uniform FBGs in silica fiber have similar thermal sensitivities, which results in a straightforward operation without complex equipment or calculations. This FBG interrogation configuration is also quite promising for dynamic measurements, and due to its multiplexing capabilities multiple USFBGs can be inscribed in the same optical fiber, allowing to incorporate several filters with identical or different spectral characteristics at specific wavelength regions in the same fiber, thus showing great potential to create and develop new sensing configurations.

## 1. Introduction

The development of fiber Bragg gratings (FBGs) began with Hill’s work on the non-linear properties of silica optical fibers doped with germanium, in which a period modulation of the core’s refractive index was achieved by introducing an interference pattern into the optical fiber core [1]. Later, important milestones were accomplished in the development and production of FBGs, such as the demonstration of a strong variation in the core´s refractive index when a silica optical fiber doped with germanium was irradiated by a periodic pattern originated by the intersection of two coherent ultraviolet (UV) beams [2], and the introduction of the phase mask for side inscription, originating a method for mass fabrication of FBGs due to the simplicity of the technique, easy alignment and repeatability [3].

Since the development of their production methods, FBGs have been increasingly studied and used as optical sensors for a variety of applications. Optical sensors have several advantages over conventional electric sensors, namely the immunity to electromagnetic interference, small size, lightweight and resistance to harsh environments. In addition, FBG-based sensors can operate in reflection and have multiplexing capabilities, which allows to photo-inscribe several FBGs along a single fiber length, creating quasi-distributed sensor arrays. Despite being one of the most used and well-known structure in optical sensing, and receiving great attention for several applications, FBGs have a major drawback regarding the cross-sensitivity effects, specially from strain and temperature variations, making it difficult to separately determine the parameter that affected the wavelength shift of a single FBG. This phenomenon can be even more problematic when working with certain coatings or/and polymer optical fibers (POFs), since other parameters such as humidity and refractive index may also take part in the cross-sensitivity effects. An example is *poly(methyl methacrylate)* (PMMA), which is widely used to produce POFs and has a high affinity to water and therefore is also sensitive to humidity variations [4], resulting in one more parameter to address to attenuate the cross-sensitivity effects. Several methods have been developed to solve this issue, most of them based on dual-grating where the different sensitivity capabilities are explored. Multiple techniques that rely on 2 gratings for parameter discrimination have been investigated, using silica fiber [5,6,7,8,9], POF [10,11,12] and even both [13]. Other alternative is the combination of an FBG with other optical fiber sensing structure, namely long period grating (LPG) [14] and interferometers [15,16,17]. The use of a single FBG for multiparameter sensing, and therefore cross-sensitivity mitigation, have also been an object of research over the years, in which some techniques have been demonstrated in silica [18,19,20] and POF [21,22].

Although the previous techniques produced reliable and innovating sensing solutions to attenuate the cross-sensitivity effects of the FBGs, most of them with the capability for multiparameter sensing allowing to differentiate different parameters, theses sensors were designed to be used through wavelength detection, which require expensive interrogation devices and, in most cases, also bulky equipment. A viable solution is converting the reflective wavelengths into reflective intensities, using an optical edge filter to interrogate the output signal from the FBG. By transforming the wavelength shift into optical power variation, the measurement can be done with only an optical power meter/photodetector, which allows to obtain a more rapid, compact, and relative low-cost sensing system. However, obtaining appropriate optical edge filters can be difficult and/or expensive. Commercial tunable optical edge filters, such as gaussian filters [23,24], are a feasible solution but are not cost-effective. Other filtering techniques rely on the use of interferometers, namely Fabry-Perot (FP) [25], which can provide a broadband optical power slope but, on the other hand, this process require the use of temperature insensitive FP filters or placing the filters in a controlled temperature environment to not compromise mechanical measurements. The use of LPGs as edge filters is also another alternative, but as the FP filters, these devices have measurement limitations due to the surrounding environmental conditions [26]. A simple and cost-effective technique to overcome the influence of temperature is the use of a second FBG that acts as the edge filter, thus having the sensor and filter with similar temperature sensitivity. Using FBGs as edge filters can be advantageous in many applications since high sensitivities can be achieved but the operation range can be very narrow. Most of the configurations that use this interrogation technique employ twin FBGs [27,28,29], with physical length of few millimeters, but other configurations have already been explored, such as the combination of narrow and wide bandwidth FBGs [30] and the use of tilted FBGs [31] as edge filters. As mentioned before, most of these configurations show very limited operation range, and tilted FBGs require special procedures to obtain wide linear ranges [31]. A possible solution to increase the operation range is the employment of smaller length FBGs, which allows to increase the full width at half-maximum (FWHM) of the reflected signal and obtain wider side slopes. Ultra-short fiber Bragg gratings (USFBGs), with grating lengths of tens or hundreds of micrometers, present some spectral characteristics, namely the broader reflection spectra, that make them suitable for filtering operations. Although their ultra-short lengths result in low reflectivity gratings, they are a cost-effective solution as optical edge filters, and their use can improve and simplify the interrogation of FBG sensors. In addition, these grating devices have already been implemented in some interrogation solutions, combining with tunable lasers [32] and optical gaussian filters [33]. Due to their spectral characteristics, they have great potential in several applications, namely in the production of FP interferometers [34] and quasi-distributed sensors [34,35,36]. 

In this paper, we propose a simple, efficient and low-cost FBG interrogation solution using USFBGs as edge filters. This principle of operation is demonstrated in silica fiber and PMMA microstructured-POF (mPOF). Since the gratings have similar thermal sensitivities, the sensor configuration can be considered temperature insensitive when both FBG and USFBG are affected by the same thermal conditions. This sensing configuration demonstrates great potential, since it does not need expensive and bulky equipment and neither complex calculations nor acquisition systems. Therefore, the main purposes of this work are the production of several USFBGs, in both silica and POF, using different inscription conditions to analyze the bandwidth and the side slopes of their reflected signal, and the investigation of simple and low-cost sensing configurations, able to attenuate the cross-sensitivity effects when FBGs are used as sensors. To our knowledge, this is the first time that USFBGs are produced in mPOF and that these gratings are interrogated by other POF Bragg gratings (POFBGs). 

## 2. Theoretical Concepts

The reflectivity is an important optical property of an FBG that allows to evaluate the “strength” of the grating in terms of effectiveness in reflecting the incident signal. According to the coupled mode theory, the reflectivity of a uniform FBG of length *L* can be described by the following equation [37]:

(1) $$R=\frac{\sinh^{2}\left(\sqrt{\kappa {\;}^{2}+{\hat{\sigma }}^{2}}L\right)}{\cosh^{2}\left(\sqrt{\kappa {\;}^{2}+{\hat{\sigma }}^{2}}L\right)-\frac{{\hat{\sigma }}^{2}}{\kappa {\;}^{2}}}$$

where *κ* is the “AC” coupling coefficient and $\hat{\sigma }$ is a “dc” self-coupling coefficient. The FBG maximum reflectivity occurs when $\hat{\sigma }$ = 0, and is given by:

(2) $$R_{max}=\tanh^{2}\left(\kappa L\right)$$

In the case of a uniform 1st order FBG in single mode (SM) optical fiber, the relationship between *κ* and the induced refractive index change (Δ*n*) is:

(3) $$\kappa =\frac{\pi \Delta n}{\lambda }$$

where *λ* is the resonant wavelength. The USFBGs are a type of “weak gratings”, which are characterized by their low reflectivity. From the previous equations, “weak gratings” can be produced by inducing a low Δ*n* and/or by very short grating lengths. Different from the conventional low Δ*n* “weak gratings”, the low reflectivity from the USFBGs usually results from the ultra-short *L* values. In addition to the reflectivity, the parameter *L* also affects the bandwidth of the reflected signal, especially for “weak gratings”. This parameter defines the total number of grating periods (N=L/Λ), and as *N* gets bigger or smaller, the reflection bandwidth becomes narrower or broader, respectively for a given value of *κL*. The relationship between *L* and the bandwidth between the first minimums on either side of the maximum reflectivity is described by [37,38]:

(4) $$\Delta \lambda =\frac{\lambda^{2}}{\pi n_{eff}L}\sqrt{{\left(\kappa L\right)}^{2}+\pi^{2}}$$

where *n_eff_* is the effective refractive index of the guided mode. For “weak gratings”, which satisfy the condition ${\left(\kappa L\right)}^{2}\ll \pi^{2}\;$, the bandwidth is an inverse function of the grating length (or parameter *N*) and can be estimated by:

(5) $$\Delta \lambda \approx \frac{\lambda^{2}}{n_{eff}L}=\frac{2\lambda }{N}$$

In the case of “strong gratings” (if ${\left(\kappa L\right)}^{2}\gg \pi^{2}$), the bandwidth can be obtained by:

(6) $$\Delta \lambda \approx \frac{\lambda^{2}\kappa }{\pi n_{eff}}=\frac{\lambda \Delta n}{n_{eff}}$$

In this type of gratings, the light may not penetrate the full length of the grating, and thus the bandwidth is independent of *L* and proportional to *κ*, and consequently Δ*n*.

## 3. Materials and Methods

### 3.1. Gratings Inscription Setup

**Figure 1:**
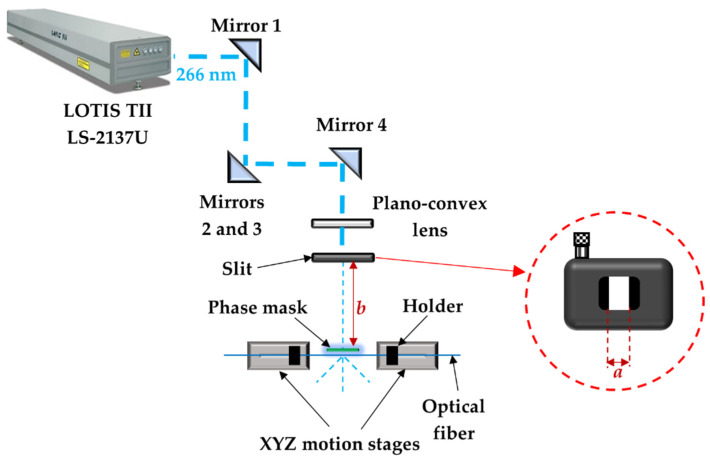
Sketch of the FBG inscription setup (*a* and *b* correspond to the slit width and the distance between the slit and the phase mask, respectively).

The FBG inscription setup, based on the phase mask method, is shown in Figure 1. The production of the FBGs in both silica fiber and POF was performed by the fourth harmonic (@266 nm) of a pulsed Q-switched Nd:YAG laser system (LOTIS TII LS-2137U Laser, Minsk, Belarus). The laser beam profile is circular, the diameter is about 8 mm and the divergence ≤1.0 mrad. The laser system has a maximum pump energy of 60 J and a maximum pulse repetition rate of 10 Hz, with a pulse duration of 8 ns. Before reaching the phase mask, the laser beam is reflected by four mirrors and then focused onto the fiber core by a plano-convex cylindrical lens with effective focal length of 320 mm, originating an effective spot size on the fiber surface with about 8 mm in width (along the fiber length) and about 30 µm in height. The laser path height increases between the mirrors 2 and 3 since the laser beam output and mirrors 1 and 2 are in a lower position in relation with the other optical components. In the production of the USFBGs, a slit was added to the system, placed between the plano-convex lens and the phase mask. During the inscription, the reflection optical spectra were monitored by an optical interrogator (Micron Optics SM-125-500, LUNA Innovations, Atlanta, Georgia).

### 3.2. USFBGs Production in Silica Fiber

The silica optical fiber used to inscribe the USFBGs was the SM GF1 fiber (Thorlabs, Newton, NJ, USA). This fiber was hydrogenated at a hydrogen pressure of 120 bar during 2 weeks to enhance its photosensitivity during the inscription process. USFBGs with different lengths were inscribed in this fiber, using a 10 mm phase mask with a pitch of 1058.04 nm. In the inscription process of each USFBG, the laser system operated with 25.5 J of pump energy and a pulse repetition rate of 10 Hz during 3 min.

### 3.3. USFBGs Production in PMMA mPOF

**Figure 2:**
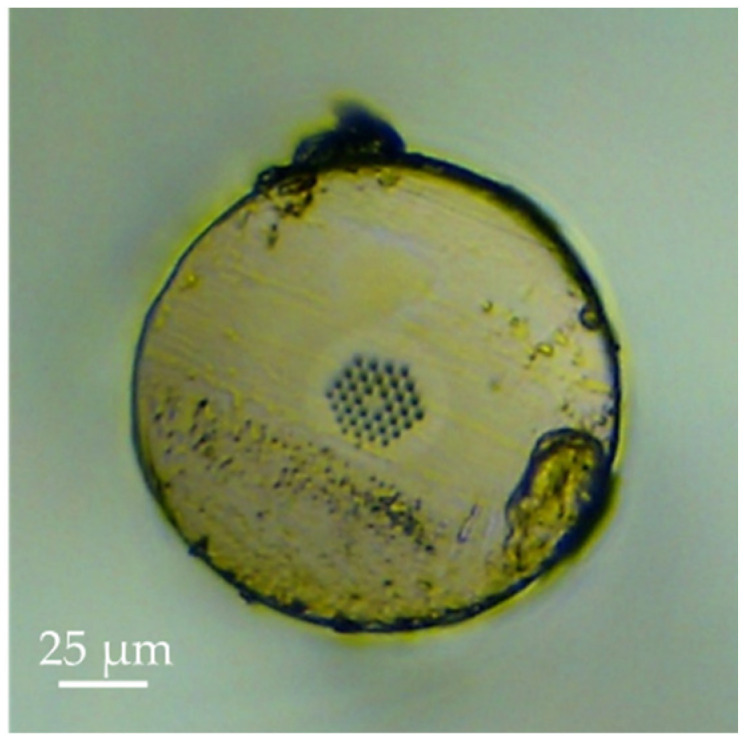
Cross-section of the 3-ring PMMA mPOF.

The production of ultra-short POF Bragg gratings (USPOFBGs) was performed to compare results with silica counterparts and promote their potential in various sensing applications, due to the POFs advantages regarding their physical properties and sensitivities [39,40,41,42]. The USPOFBGs were inscribed in 3-ring hexagonal hole structure undoped PMMA POF (see Figure 2), manufactured at DTU Electro (Lyngby, Denmark). The outer diameter, hole diameter and pitch of the endless single mode mPOF are 130 μm, 1.90 μm and 4.60 μm, respectively. The mPOF samples were pre-annealed at 70 °C during 24 h, glued to ferrule connectors and later cleaved with a hot blade. The inscription setup was the same used to produce silica USFBGs, and the laser system parameters were 25 J of pump energy and pulse repetition rate of 10 Hz. The pitch of the 10 mm phase mask employed on the production of the USPOFBGs is 1053.90 nm and the inscription time was about 10 min. Due to the high attenuation of the PMMA material in the 1500 nm region, the gratings were inscribed close to the connector, located approximately 40 mm from the cleaved end face, and index matching gel was applied between the silica fiber and the mPOF connectors to avoid signal reflections and/or the formation of Fabry-Perot cavities.

### 3.4. Sensing Configuration Description

**Figure 3:**
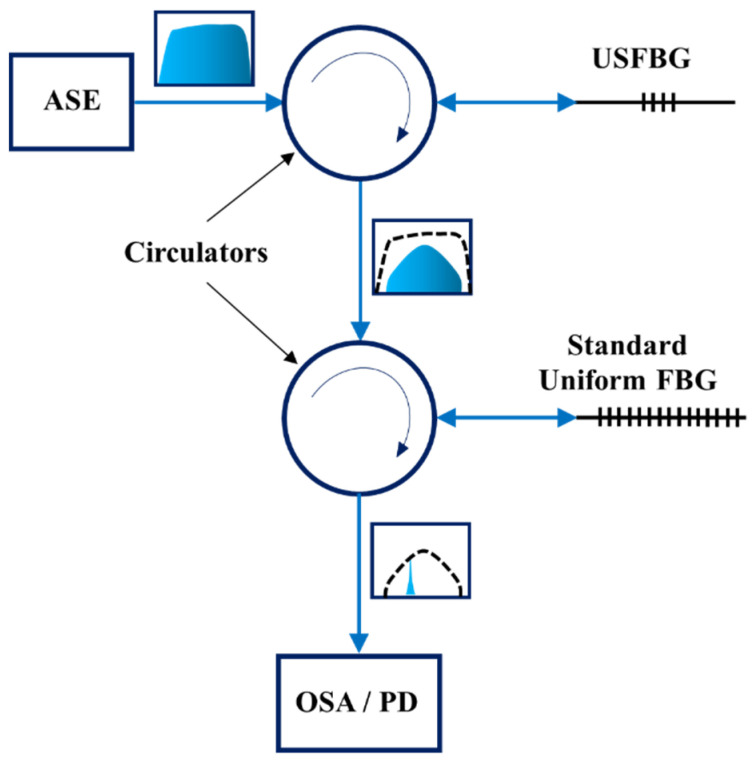
Schematic representation of the setup used to interrogate a uniform FBG, employing a USFBG as the edge filter.

The basic schematic and functionality of the interrogation and sensing system is depicted in Figure 3. In its simpler and cost-effective way, this configuration comprises an optical broadband source, two circulators, an USFBG, a uniform FBG and a Photodetector (PD). The light from the broadband source is sent to the USFBG by an optical fiber circulator, where the reflected signal from this grating is directed to the FBG by a second circulator. Thus, the resulting signal is a correlation between the reflected spectra from both USFBG and the uniform FBG, and its optical power is detected by the PD. 

To avoid measurement errors due to optical power fluctuations from the broadband source, an optical coupler can be added to the configuration, in which a portion of the signal goes to another PD (reference) and the remaining continue the path of Figure 3. Its location can be before the USFBG, to monitor power fluctuations of the light from the broadband source, or after the USFBG, to monitor fluctuations of the signal reflected by the USFBG, before reaching the uniform FBG. The importance of the use of a reference is related to the stability of the employed broadband source. In addition, the type of measurement (static/dynamic) and to the amount of acceptable measurement errors are important factors when it is necessary to evaluate the use of a reference or not. In this work, the reference was not used, since the purpose is to characterize the use of USFBGs as edge filter to interrogate a uniform FBG and also due to the relatively good stability of the Amplified Spontaneous Emission (ASE) broadband light source (AS4500 Series from Shanghai B&A Technology, Shanghai, China) employed in this configuration. Additionally, in order to perform a spectral analysis to the resulting signal, an Optical Spectrum Analyzer (OSA) was used instead of the PD. 

## 4. Results and Discussion

### 4.1. Silica USFBGs

**Figure 4:**
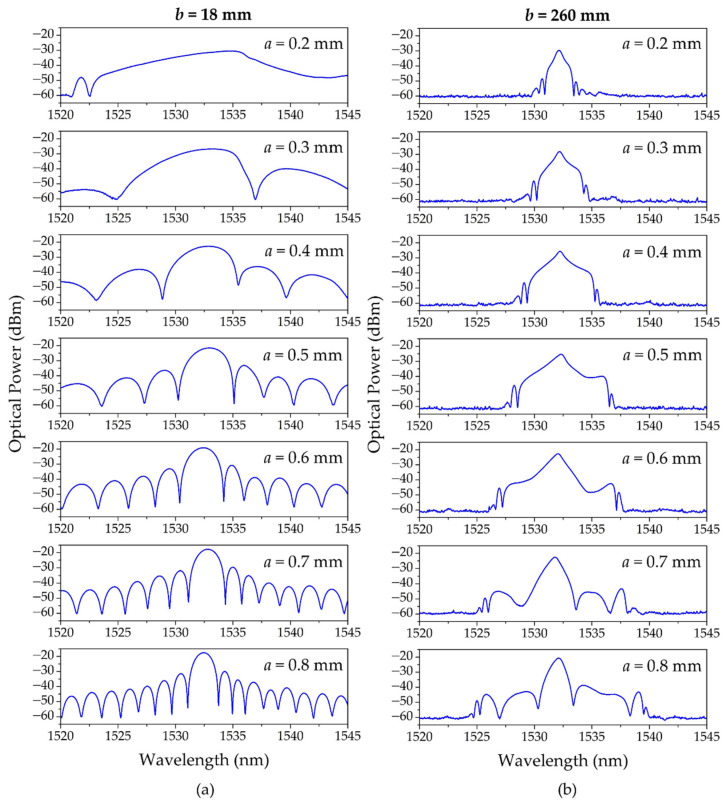
Reflected spectra of the produced USFBGs with different slit widths when: (**a**) *b* = 18 mm; (**b**) *b* = 260 mm.

The first USFBGs were inscribed with the slit fixed at 18 mm from the phase mask (closer position to the phase mask in this setup), using different slit widths (*a*): 0.2, 0.3, 0.4, 0.5, 0.6, 0.7 and 0.8 mm. Figure 4a shows the reflection spectra of the produced USFBGs. These results are in accordance with the theoretical concepts, as the bandwidth of the reflected signal increases as the length of the gratings decreases. Other observable characteristic of the spectra from Figure 4a is the high amplitude of the sidelobes, resulted from absence of apodization of the refractive index change on the limits of the gratings. This phenomenon is originated by the combination of two parameters: very small distance between the slit and the phase mask (*b*) and the small *a* values used, in which only small portions of the center of the laser beam diameter reached the phase mask. Both parameters are related to the diffraction patterns of the laser beam towards the phase mask, and consequently they affect the grating physical length, bandwidth of the reflective spectrum, and the presence (and amplitude) of the sidelobes [43,44].

Alternatively, USFBGs were produced in the same optical fiber using the same inscription setup, except the *b* value, which was adjusted to 260 mm (higher *b* value in this setup). Figure 4b presents the reflective spectra of the produced USFBGs, with different *a* values. Comparing with the results from Figure 4a, the optical characteristics of the reflection signal vary substantially for the same slit widths, namely the bandwidth and the suppression of sidelobes. The results demonstrate that decreasing the slit width to obtain smaller length USFBGs, and consequently generate a broader reflection bandwidth, does not occur for small slit apertures in this new experimental configuration (*b* = 260 mm). In fact, it occurs exactly as the opposite effect, as *a* decreases (specially for values lower than 0.6 mm), the bandwidth decreases proportionally. Despite being used to control the size of the UV laser beam to produce gratings with the desired length as *a* becomes smaller, diffraction effects take place and become more significant. Therefore, for very small aperture dimensions, the USFBGs length are no longer proportional to *a*. In addition to *a*, parameter *b* is also important when considering the diffraction effects, allowing to analyze if the diffraction is near field (Fresnel diffraction) or far field (Fraunhofer diffraction). A simple way to define the diffraction regime is by the Fresnel number (*N_F_*), which is given by:

(7) NF=a22bλUV

where *λ_UV_* is the incident wavelength of the UV laser beam, which, in this case, is 266 nm. For *N_F_* >> 1, the diffraction pattern is near field, while for *N_F_* << 1 (Fraunhofer Condition), the diffraction pattern is far field. The Fraunhofer diffraction pattern can become much wider than the slit width and in the USFBG inscription process by reducing the slit width to produce smaller length gratings, the opposite occurs for this diffraction regime. On the other hand, as the *N_F_* increases (by increasing *a* and/or decreasing *b*), the diffraction pattern size approaches the size of the slit width [43]. 

**Figure 5:**
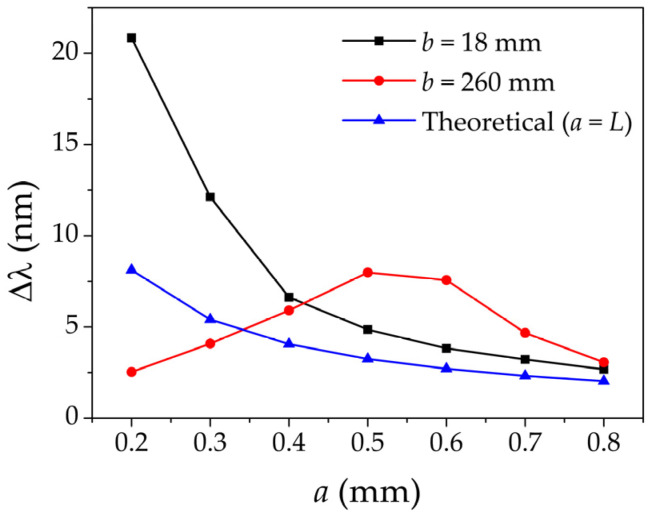
Bandwidth variation as function of the slit widths for *b* values of 18 mm and 260 mm and as function of the gratings length, *L* (for *λ* = 1532 nm and *n_eff_* = 1.445).

The information in Figure 5 and Table 1 demonstrates the previous statements. For the proposed slit widths, as *b* takes the value of 18 mm, *N_F_* > 1 and the diffraction pattern can be considered near field, particularly for higher values of *a*, as their bandwidth approaches the theoretical values when the grating length is equal to the slit width. On the other hand, when *b* is 260 mm, *N_F_* < 1, and when *a* ≤ 0.5 mm, the diffraction pattern approaches the far field regime, since the bandwidth increases as function of *a*. For *a* ≥ 0.6 mm, *N_F_* > 1 and the bandwidth begins to decrease as function of *a*, approaching the theoretical values when *a* = *L*. Table 1 also shows the 3 dB bandwidth for the produced USFBGs, which varies almost according to the bandwidth variation from Figure 5. For *b* = 18 mm, the smaller 3 dB bandwidth is 1.195 nm (*a* = 0.8 mm) and the larger is 5.195 nm (*a* = 0.2 mm), while, for *b* = 260 mm, the smaller and larger 3 dB bandwidths are 0.580 nm (*a* = 0.2 mm) and 0.935 nm (*a* = 0.5 mm), respectively.

### 4.2. PMMA mPOF USFBGs

**Figure 6:**
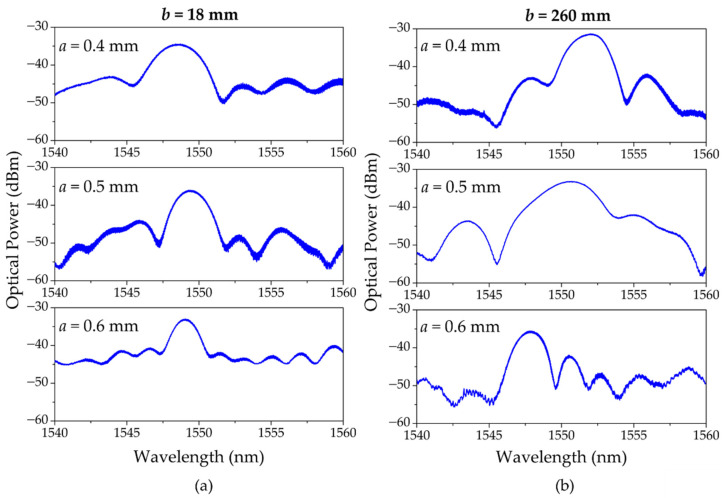
Reflected spectra of the produced USPOFBGs with different slit widths when (**a**) *b* = 18 mm; (**b**) *b* = 260 mm.

**Figure 7:**
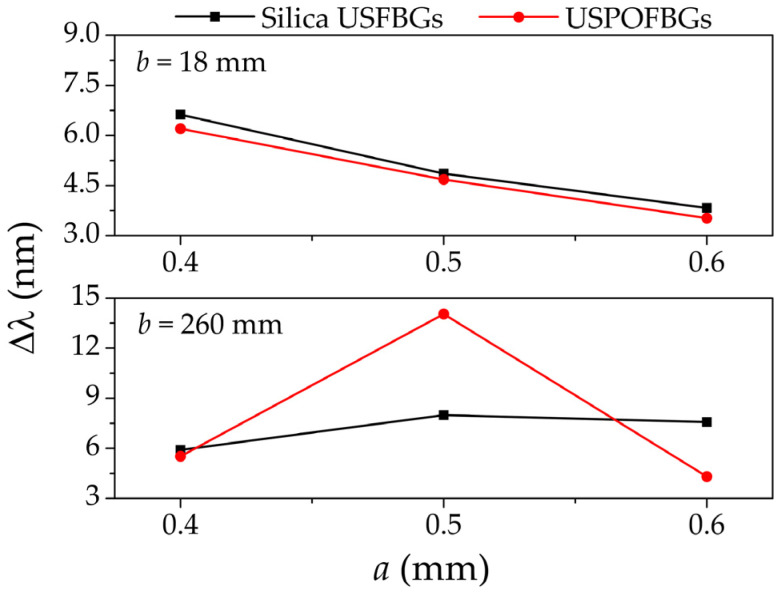
Bandwidth variation as function of the slit widths for silica USFBGs and USPOFBGs when *b* is 18 mm and 260 mm.

After the proof of concept from the production of USFBGs in silica fiber, the slit widths used to produce the USPOFBGs were 0.4 mm, 0.5 mm and 0.6 mm. The reflection spectra of these gratings, inscribed when *b* = 18 mm and *b* = 260 mm, are depicted in Figure 6a,b, respectively. Once again, the reflection spectra from the obtained USPOFBGs demonstrate the differences regarding the diffraction pattern (and consequently the inscription process) and the value of *b*. Figure 7 compares the bandwidth of the produced USFBGs in silica and polymer fiber, when *b* is 18 mm and 260 mm. In the case of *b* = 18 mm, the diffraction pattern is considered near field, and the bandwidth of both silica USFBGs and USPOFBGs are similar and decrease as *a* increases. On the other hand, for *b* = 260 mm, the results show some discrepancies between the silica and polymer gratings bandwidths, which (besides other factors that may affect the inscription) is result of a less defined diffraction pattern for those *a* values, as the *N_F_* are relative close to 1. In addition, while in the silica USFBGs the reflection spectral characteristics were identical when multiple samples were produced under the same inscription conditions, in the USPOFBGs, small differences were observed between some of the samples regarding the reflection optical power and bandwidth. This can be explained by the presence of the microstructure holes in the laser path, which may result in light scattering, affecting the inscription efficiency and possibly the gratings physical length. Despite the existence of those differences in the inscribed USPOFBGs, the evolution of the bandwidth in function of the parameters *a* and *b* follows the behavior described in Figure 7. The largest bandwidth and 3-dB bandwidth in mPOF gratings were 14.040 nm and 3.495 nm, respectively, obtained for *a* = 0.5 mm and *b* = 260 mm. 

### 4.3. Sensing Characterization

#### 4.3.1. Strain Response

**Figure 8:**
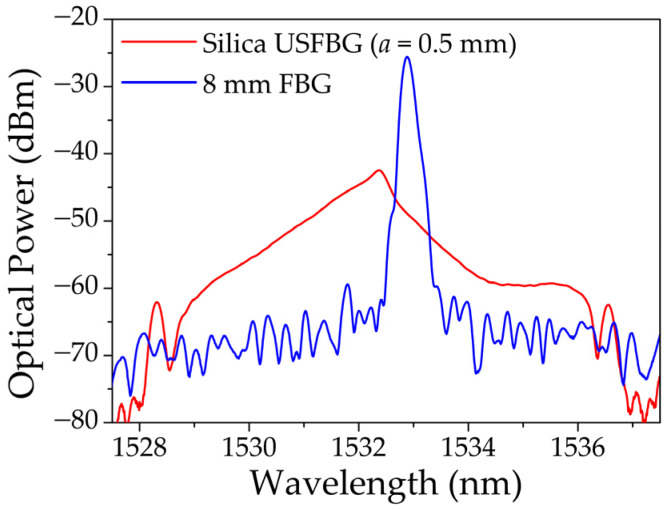
Reflection spectra of the 8 mm FBG and USFBG in silica fiber employed in the sensing tests.

To assemble the interrogation setup depicted in Figure 3 (Section 3.4), a uniform FBG, 8 mm in length, was produced in the same hydrogenated SM GF1 fiber as the USFBGs were in Section 4.1, using the same inscription system without the slit. The inscription was performed with the same laser parameters and phase mask (pitch of 1058.04 nm) as the silica USFBGs but with an inscription time of 2 min. The reflection spectrum of the produced 8 mm FBG is shown in Figure 8, together with the reflection signal from an USFBG, produced when *b* = 260 mm and *a* = 0.5 mm. These spectra were obtained using the ASE, a circulator and the OSA (model MS9740A, Anritsu, Atsugi, Japan). The 8 mm FBG has a central wavelength at 1532.880 nm, and the amplitude of the reflection peak is approximately 44 dB, while the peak wavelength and the amplitude of the reflection signal of the USFBG are 1532.370 nm and approximately 30 dB, respectively. The USFBG in Figure 8 was chosen due to the linear and relative long slope (left side), important for edge filtering and sensing capabilities. Nevertheless, other USFBGs with other spectral characteristics can be more suitable as edge filters for specific operations when there is the need to have less or more tilted edges and/or shorter or longer slopes.

The strain characterization was carried out by attaching each fiber (one with the FBG and the other with the USFBG) to a fixed and a manual translating stage, with 10 µm resolution. The gratings were in the same room, subjected to the same temperature, and the testing configuration demonstrated in Figure 3 was used, where the resulting optical signal was monitored with the OSA. Since both gratings can be used as strain sensors, the tests were performed in two phases: first the USFBG was fixed and strain was applied in the uniform FBG, and in the second phase the uniform grating was fixed and the USFBG was subjected to strain. 

**Figure 9:**
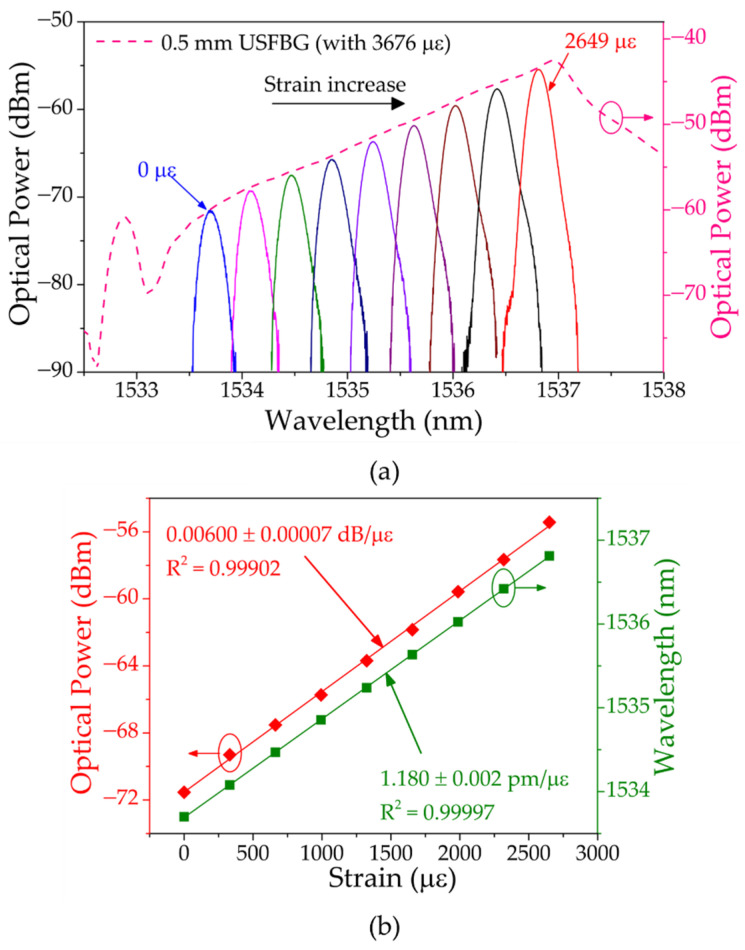
(**a**) Reflection spectra of the resulting signal at different levels of strain on the uniform FBG fiber and of the USFBG with 3676 με. (**b**) Corresponding wavelength and optical power shift with increasing strain.

In the first phase, 3676 με was applied to the USFBG to red shift the grating reflection signal and for the uniform FBG go through the entire left slope of USFBG. Figure 9a shows the spectra of the resulting signal, when applying different levels of strain on the uniform FBG, in which is possible to observe both the wavelength and optical power shift along the left slope of the strained USFBG. With steps of 331 με, the fiber was stretched until the FBG wavelength passed through the USFBG peak. The last strain value measured on the left slope of the USFBG was 2649 με, resulting in a wavelength tuning of 3.1 nm and a total optical power variation of 16.24 dB. The wavelength and optical power shift with the increasing strain is presented in Figure 9b, and the obtained sensitivities were 1.180 ± 0.002 pm/με and 0.00600 ± 0.00007 dB/με, respectively. The results show a good linearity of the optical power variation, which resulted from the linear left slope of this USFBG, with a coefficient of determination (R^2^) of 0.99902.

**Figure 10:**
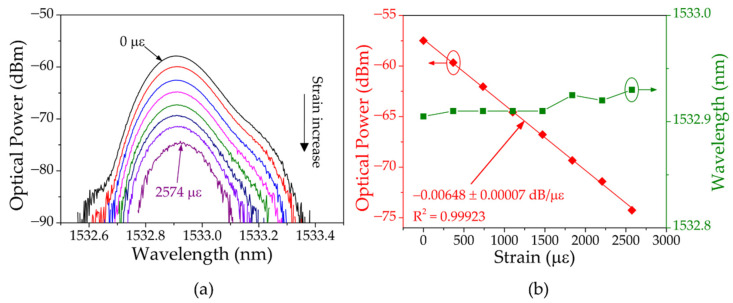
(**a**) Reflection spectra of the resulting signal at different levels of strain on the USFBG fiber. (**b**) Corresponding wavelength and optical power shift with increasing strain.

In the second phase, the uniform FBG was kept unstrained, while the USFBG was stretched with steps of 368 με. The strain measurement began once the Bragg wavelength of the uniform FBG was positioned in the USFBG left slope wavelength range. The spectra of the resulting signal for different levels of strain on the USFBG is presented in Figure 10a, and the corresponding wavelength and optical power response are given by Figure 10b. Dissimilar to the first phase, where the FBG swept the USFBG left slope from the bottom to the top with the increasing strain, in this case, in the beginning of the strain measurement, the FBG wavelength is located at the top of the slope, and as the strain increases on the USFBG fiber, the slope shifts to higher wavelengths, leading to the decrease of the optical power in the resulting signal (see Figure 10a). Additionally, in this scenario, the wavelength of the resulting signal is almost constant, with a maximum variation of 25 pm when the strain on the USFBG was increased up to 2574 με, at which point the FBG wavelength was located at the bottom of the slope. On the other hand, the variation of the optical power was linear with an obtained sensitivity of −0.00648 ± 0.00007 dB/με and a R^2^ of 0.99923.

**Figure 11:**
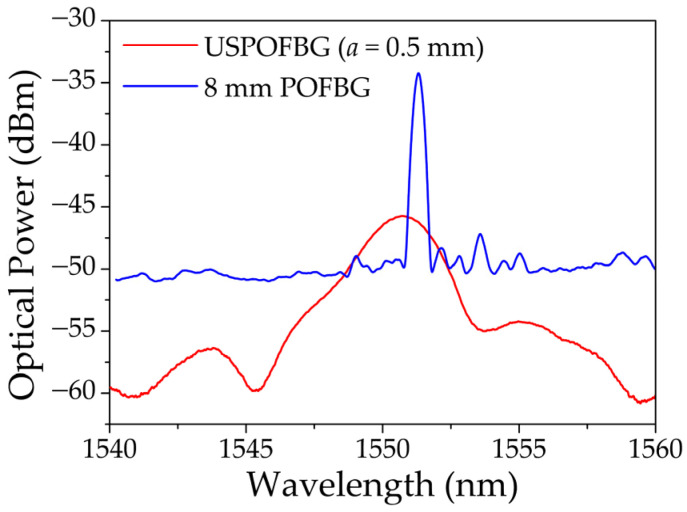
Reflection spectra of the 8 mm POFBG and USPOFBG employed in the sensing tests.

The procedure and setup to characterize the strain response and filtering capabilities of the mPOF gratings is the same as the one used to test the silica fiber gratings. An 8 mm POFBG was also inscribed in the annealed three-ring undoped PMMA mPOF, located about 40 mm from the cleaved end face in connector. The inscription setup was the same, described in Section 3.1 (without the slit), and the procedure, laser parameters and phase mask pitch were identical to the ones used to inscribe the USPOFBGs. The reflection spectra, measured with the OSA, of the produced 8 mm POFBG and the USPOFBG, with *b* = 260 mm and *a* = 0.5 mm, are depicted in Figure 11. The central wavelengths of the 8 mm POFBG and USPOFBG are 1551.310 nm and 1550.730 nm, respectively, and the amplitude of the reflection peaks are approximately 16 dB and 15 dB, respectively. The small peaks and fluctuations around the main FBG reflection peak could be due to an imperfect cleave of the mPOF, which are well known to be difficult to cleave [45] and due to the fact that the splice is between two different geometry fibers.

**Figure 12:**
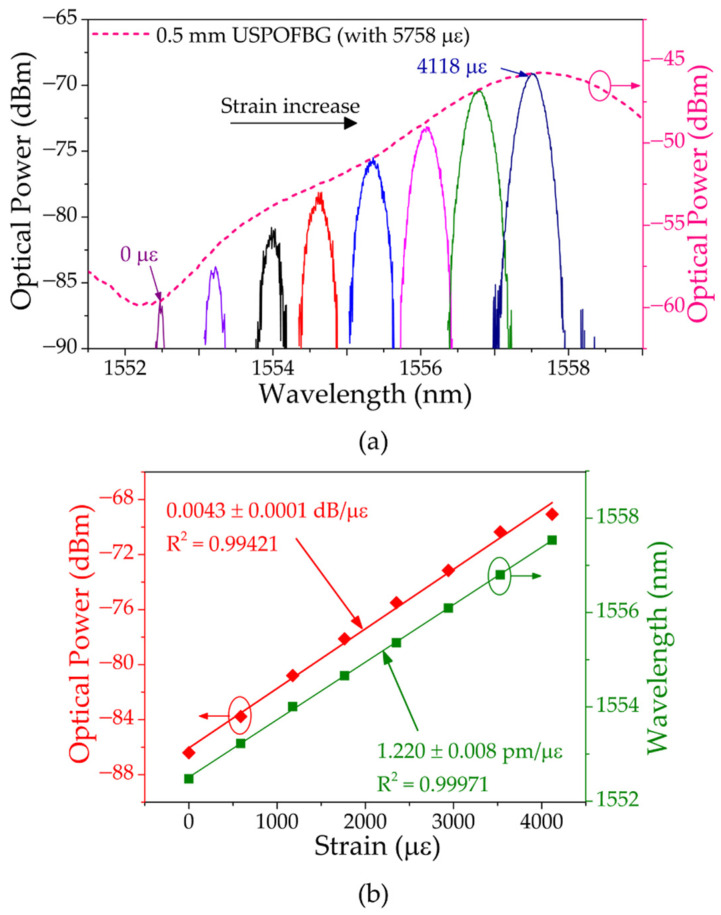
(**a**) Reflection spectra of the resulting signal at different levels of strain on the uniform POFBG fiber and of the USPOFBG with 5758 με. (**b**) Corresponding wavelength and optical power shift with increasing strain.

Just the same as the silica counterparts, the polymer gratings, which were also in the same room and subjected to the same temperature, were subjected to strain in two phases. In the first phase, the USPOFBG was fixed at 5758 με, and later, different strain levels were applied in the uniform POFBG to go through the entire left slope of USPOFBG. The spectra of the resulting signal and its position on the left slope of the strained USPOFBG with the increasing strain is presented in Figure 12a. The uniform POFBG fiber was stretched, with steps of 588 με, until the wavelength reached the USPOFBG peak. The total amount of strain applied to the uniform POFBG wavelength sweep through the left slope of USPOFBG from the bottom to the top was 4118 με, which resulted in a wavelength tuning of 5.0 nm and a total optical power variation of 15.67 dB. The wavelength and optical power shift with the increasing strain is presented in Figure 12b, and the obtained sensitivities after linearization were 1.220 ± 0.008 pm/με and 0.0043 ± 0.0001 dB/με, respectively, with R^2^ values of 0.99971 and 0.99421, respectively. 

**Figure 13:**
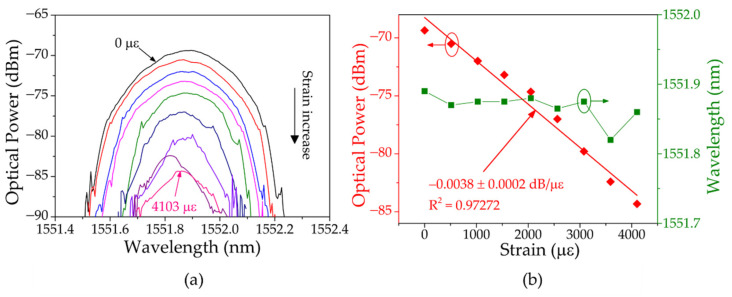
(**a**) Reflection spectra of the resulting signal at different levels of strain on the USPOFBG fiber. (**b**) Corresponding wavelength and optical power shift with increasing strain.

In the second phase, the uniform POFBG was fixed and the USPOFBG was subjected to strain variations using steps of 513 με. The strain measurement started when the POFBG Bragg wavelength was located at the top of the USPOFBG left slope. The position of the POFBG Bragg wavelength on the slope varied from the top to bottom, as the USPOFBG reflection signal shifted to higher wavelengths with the increasing strain. This led to the decrease of the optical power in the resulting signal and the almost stagnation of its wavelength, as Figure 13a demonstrates, when the USPOFBG was strained at different levels. The corresponding wavelength and optical power are presented in Figure 13b, which, after increasing the strain up to 4103 με (when the POFBG wavelength was located at the bottom of the slope), the total variation was 70 pm and 14.97 dB, respectively. After linearization, the sensitivity related to the optical power variation was −0.0038 ± 0.0002 dB/με, with a R^2^ of 0.97272.

#### 4.3.2. Temperature Response

**Figure 14:**
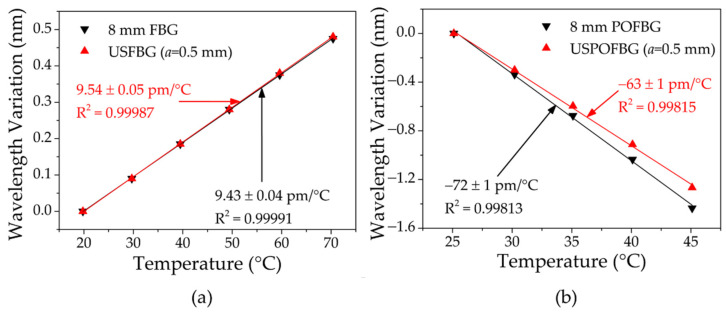
Wavelength variation of the 8 mm FBG and USFBG with increasing temperature: (**a**) in GF1 fiber; (**b**) in 3-rings undoped PMMA mPOF.

Temperature characterization was performed with the same gratings used in the strain tests to evaluate any thermal sensitivity discrepancy between the uniform FBGs and the USFBGs. In the first instance, the thermal response of each grating was analyzed, using a climate chamber (Weiss Technik LabEvent, model L C/64/70/3, Weiss Umwelttechnik GmbH, Reiskirchen, Germany) with 0.1 °C resolution. During all these measurements, to ensure thermal stabilization, the temperature was kept constant for 30 min in each step. For the silica fiber gratings, the temperature was increased from 20 °C up to 70 °C, using steps of 10 °C, without humidity control. Figure 14a shows the wavelength variation during this trial of the uniform FBG and the USFBG. After the linear fit, the temperature sensitivities were 9.43 ± 0.04 pm/°C and 9.54 ± 0.05 pm/°C for the 8 mm FBG and USFBG, respectively. These sensitivity values are almost identical to the point that the small difference between them can be associated to measurement errors and/or equipment resolution. The results demonstrate that, for this range of temperature changes, the wavelength variation is the same for both gratings; therefore, both the sensor (FBG) and edge filter possess similar temperature sensitivities. For the PMMA mPOF gratings, the temperature was increased from 25 °C up to 45 °C, using steps of 5 °C, and a fixed relative humidity (RH) of 50%. The wavelength variation of the uniform POFBG and the USPOFBG during this thermal test is shown in Figure 14b, and the obtained sensitivities were −72 ± 1 pm/°C and −63 ± 1 pm/°C, respectively. In this case, and dissimilar to the silica fiber gratings, the temperature sensitivity of the USPOFBG is about 9 pm/°C lower than the 8 mm POFBG, even though the mPOF, the thermal treatment before inscription (described in Section 3.3), the gratings spectral region and the inscription laser system are the same. A possible explanation could be related to the different inscription conditions between the gratings (the diffraction regime imposed by parameters *a* and *b* affect the laser beam intensity on the fiber during the production of the USFBGs) and, consequently, the photochemical processes induced in the PMMA structure by different irradiation energy densities [46,47,48,49]. However, further investigation is needed to analyze this behavior. 

**Figure 15:**
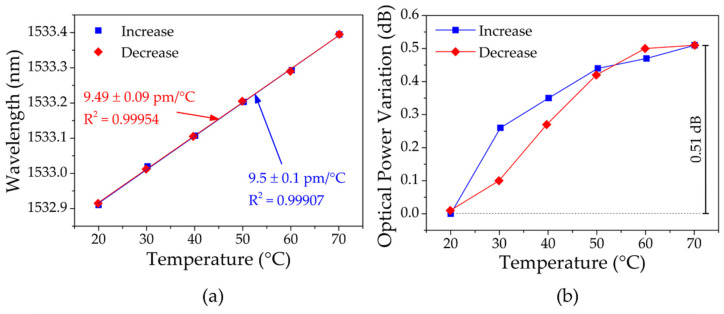
Resulting signal response from the silica gratings to the increasing/decreasing temperature: (**a**) wavelength; (**b**) optical power variation.

The next step was to evaluate the resulting signal of the experimental setup from Figure 3 under the influence of temperature changes. The uniform FBG and the USFBG fibers were placed unstrained together inside the climate chamber and, therefore, were under the same thermal conditions. Regarding the silica gratings, the temperature was once again increased from 20 °C up to 70 °C and later decreased back to 20 °C, with steps of 10 °C. The wavelength shift and the optical power variations of the resulting signal in the function of temperature are presented in Figure 15a,b, respectively. The wavelength sensitivities obtained were 9.5 ± 0.1 pm/°C and 9.49 ± 0.09 pm/°C for increasing and decreasing temperatures, respectively, which is in accordance with the ones obtained previously in both silica fiber uniform FBG and USFBG. During the test, the optical power registered a maximum variation of 0.51 dB, which is a satisfactory value since several factors can contribute to the optical power level oscillations, since the fluctuations on the ASE and the connections between fibers and equipment to minor influences of temperature in the grating reflectivity and, therefore, in the reflective optical power. 

**Figure 16:**
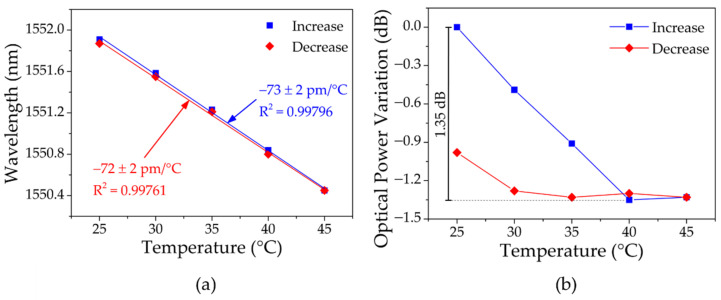
Resulting signal response from the PMMA mPOF gratings to the increasing/decreasing temperature: (**a**) wavelength; (**b**) optical power variation.

The resulting signal from the setup with the mPOF PMMA gratings was monitored for increasing/decreasing temperature in the range 25–45 °C using 5 °C steps and 50% RH. Figure 16a presents the wavelength shift, which showed thermal sensitivities of −73 ± 2 pm/°C (temperature increase) and −72 ± 2 pm/°C (temperature decrease). The optical power variation with temperature is demonstrated in Figure 16b, showing a sharp descent when the temperature increased to 40 °C of approximately 1.35 dB. Among the possible factors already mentioned before that contribute to the optical power variations, which, in the case of the fiber connections and reflectivity stability, may be enhanced when using POFs, the difference in the thermal sensitivities between the uniform POFBG and the USPOFBG contributed even further to these results. In fact, operating with PMMA mPOFs in this configuration can be challenging, especially in the 1550 nm region due to the high attenuation of this polymer at these wavelengths, which, combined with POF-silica optical fiber connection losses, leads to weaker resulting signals when compared with the silica counterparts.

## 5. Conclusions

In this work, USFBGs with different dimensions in silica optical fiber were produced and PMMA mPOF and demonstrated that the manipulation of the slit aperture and the distance between the slit and the phase mask can lead to the production of gratings with desirable filtering characteristics. It was demonstrated that the USFBGs produced under a diffraction regime with a Fresnel number around 1 present not only self-apodization but also unique spectral characteristics with great potential for edge filtering applications. With these devices, it was proposed a simple, efficient and low-cost FBG interrogation solution using USFBGs as edge filters, and in the case of silica fiber, this configuration is almost temperature insensitive, since both USFBG and uniform FBG have similar thermal sensitivities. Regarding the mPOF gratings thermal performance, an approximately 9 pm/°C difference between the USFBG and the uniform FBG temperature sensitivities was obtained. Although this is troublesome to discriminate the temperature effects in certain applications, it does not have major effects for dynamic measurements or in relative controlled climate environments. In addition to the similar sensitivities, the use of USFBGs brings additional advantages related to the usage of a relative low-cost tunable edge filter, which can be produced at specific wavelengths and with different linear edge slopes, the capability of both FBG and USFBG being used as sensing and interrogating elements and the capability of incorporate multiple filters in a single fiber due to the multiplexing capabilities of these devices. Thus, this configuration shows great potential due to its simplicity, straightforward operation, cost-effectiveness and possibility to evolve and be used in several applications. 

## Figures and Tables

**Table 1:** *N_F_* and 3 dB bandwidth values of the produced USFBGs.

*a* (mm)	*b* = 18 mm		*b* = 260 mm	
	*N_F_*	3-dB Bandwidth (nm)	*N_F_*	3-dB Bandwidth (nm)
0.2	2.089	5.195	0.145	0.580
0.3	4.699	4.375	0.325	0.645
0.4	8.354	2.965	0.578	0.720
0.5	13.054	2.170	0.904	0.935
0.6	18.797	1.725	1.301	0.855
0.7	25.585	1.430	1.771	0.810
0.8	33.417	1.195	2.314	0.785

## Data Availability

The data presented in this study are available on request from the corresponding author.
